# Chimeric TALE recombinases with programmable DNA sequence specificity

**DOI:** 10.1093/nar/gks875

**Published:** 2012-09-26

**Authors:** Andrew C. Mercer, Thomas Gaj, Roberta P. Fuller, Carlos F. Barbas

**Affiliations:** The Skaggs Institute for Chemical Biology and the Departments of Molecular Biology and Chemistry, The Scripps Research Institute, La Jolla, CA 92037, USA

## Abstract

Site-specific recombinases are powerful tools for genome engineering. Hyperactivated variants of the resolvase/invertase family of serine recombinases function without accessory factors, and thus can be re-targeted to sequences of interest by replacing native DNA-binding domains (DBDs) with engineered zinc-finger proteins (ZFPs). However, imperfect modularity with particular domains, lack of high-affinity binding to all DNA triplets, and difficulty in construction has hindered the widespread adoption of ZFPs in unspecialized laboratories. The discovery of a novel type of DBD in transcription activator-like effector (TALE) proteins from *Xanthomonas* provides an alternative to ZFPs. Here we describe chimeric TALE recombinases (TALERs): engineered fusions between a hyperactivated catalytic domain from the DNA invertase Gin and an optimized TALE architecture. We use a library of incrementally truncated TALE variants to identify TALER fusions that modify DNA with efficiency and specificity comparable to zinc-finger recombinases in bacterial cells. We also show that TALERs recombine DNA in mammalian cells. The TALER architecture described herein provides a platform for insertion of customized TALE domains, thus significantly expanding the targeting capacity of engineered recombinases and their potential applications in biotechnology and medicine.

## INTRODUCTION

The ability of proteins to recognize DNA in a sequence-dependent manner is central to life. A variety of protein domains have evolved to provide sequence-specific DNA recognition. DNA recognition by a select few of these domains is also the foundation for a wide variety of biotechnological applications. In particular, C_2_H_2_ zinc-finger proteins (ZFPs) were among the first DNA-binding proteins to be engineered to recognize user-defined DNA sequences and have been used with varying degrees of success for many applications, including transcriptional regulation, genome engineering and epigenetic modification (1–20). Modular assembly of ZFPs has facilitated these approaches. However, despite the advances and promise of ZFP technology, construction of specific, high-affinity ZFPs for certain sequences remains difficult and in select cases requires the use of time-consuming and labor-intensive selection systems not readily adopted by non-specialty laboratories (21).

Transcription activator-like effector (TALE) domains are a class of naturally occurring DNA-binding domains (DBDs) that represent a potential alternative to ZFP technology (22,23). TALEs, which are found in the plant pathogen *Xanthomonas*, contain a series of 33–35 amino acid repeats that function to selectively bind target DNA sequences. These repeats are identical with the exception of two adjacent repeat variable di-residues (RVDs) that confer DNA specificity by mediating binding to a single nucleotide. Arrays of over 30 repeats have been described that bind to DNA sites of similar numbers of base pairs (bps). Although there is inherent degeneracy in the binding of each RVD, recent reports have indicated that synthetic TALE proteins are specific enough to target single loci within the human genome (24,25).

The introduction of DNA double-strand breaks (DSBs) by chimeric nucleases, such as zinc-finger nucleases (ZFNs) can be used to knockout gene function or in the presence of exogenously added DNA drive cassette integration at the targeted loci (8,15,17,26). ZFNs have been extensively studied over the last decade and in some cases are approaching clinical use for gene therapy. Recently, a number of groups have explored the use of TALE DBDs fused to nucleases (TALENs) for targeted genome editing. Indeed, much of the work with ZFNs has been replicated with TALENs, as TALEs may have advantages over ZFNs in regards to DNA-binding modularity (25,27–36). However, despite impressive research with ZFNs and TALENs, questions remain about their safety and specificity. In particular, off-target cleavage events remain difficult to detect, as the most likely result of an off-target DSB is the introduction of small insertions or deletions. Additionally, repair of DSBs relies on cell machinery that varies with cell type.

An alternate approach for achieving targeted genomic modifications is the use of site-specific recombinases (SSRs). SSRs, such as the tyrosine recombinases Cre and Flp, are valuable molecular biology tools that are routinely used to manipulate chromosome structure inside cells. Because these enzymes rely on a number of complex protein–protein and protein–DNA interactions to coordinate catalysis, SSRs exhibit remarkable target site specificity. To date, however, altering the specificity of many SSRs has proven difficult (37,38). Serine recombinases of the resolvase/invertase type provide a versatile alternative to tyrosine recombinases for genome engineering. In nature, these enzymes function as multi-domain protein complexes that coordinate recombination in a highly modular manner. However, mutants of several serine recombinases have been identified that do not require accessory factors for recombination. Additionally, numerous studies have shown that the native DBDs of serine recombinases can be replaced with custom-designed ZFPs to generate chimeric zinc-finger recombinases (ZFRs) (18,19,39–41). In principle, ZFRs capable of recognizing an extended number of sequences could be generated, however, the lack of zinc-finger domains capable of recognizing all possible DNA triplets limits the potential modular targeting capacity of these enzymes. Use of custom-designed TALEs capable of recognizing virtually any DNA sequence may offer a potential solution to this problem.

In this study, we provide the first example of a TALE recombinase (TALER). Using a library of incrementally truncated TALE domains, we identify an optimized TALER architecture that can be used to recombine DNA in bacterial and mammalian cells. Any customized TALE repeat array can be inserted into the TALER architecture described herein, thus dramatically expanding the targeting capacity of engineered recombinases for applications in biotechnology and medicine (42,43).

## MATERIALS AND METHODS

### Reagents

All enzymes were purchased from New England BioLabs unless otherwise indicated. Primer sequences are provided in Supplementary Table S1.

### Plasmid construction

In order to introduce a *Bam*H1 restriction site either 5′ or 3′ to the Gin coding sequence, the Gin catalytic domain was PCR amplified with primers 5′ Gin_N-term and 3′ Gin_N-term or 5′ Gin_C-term and 3′ Gin_C-term, respectively. PCR products were ligated into the *Sac*I and *Xba*I restriction sites of pBluescriptII (Fermentas) to generate pB-Bam-Gin and pB-Gin-Bam. To generate the C-terminal and N-terminal TALER fusions, the AvrXa7 gene (kindly provided by Dr B. Yang, Iowa State University) was released from pWAvrXa7 with *Bam*H1 and ligated into *Bam*H1 sites of pB-Bam-Gin and pB-Gin-Bam (41) to establish pB-Avr-Bam-Gin and pB-Gin-Bam-Avr, respectively. Correct construction of each TALER was verified by sequence analysis (Supplementary Data).

To generate N-terminal truncations of AvrXa7, AvrXa7 was PCR amplified using the Expand High Fidelity PCR System (Roche) with 5′ Avr-n-(1-10) and 3′ Avr +28 or 3′ Avr +95 primers with the following program: 1 cycle of 3 min at 94°C, 16 cycles of 1 min at 94°C, 1 min at 52°C, 6 min at 68°C; and a final cycle of 1 h at 68°C. The Gin catalytic domain was PCR amplified under standard PCR conditions with 5′ Gin_C-term and 3′ GinNTalPCRFus and fused to truncated AvrXa7 variants by overlap PCR using the PCR conditions described above. Purified Gin-Avr PCR products were mixed in an equimolar ratio and digested with *Sac*I and *Xba*I.

To generate designer TALEs, we used the TALEN kit (Addgene) described by Cermak *et al.* (27) with the following modification: pTAL1 was modified to include truncations at Δ120, Δ128 or +28. To achieve this, AvrXa7Δ120 and AvrXa7Δ128 fragments were PCR amplified with 5′ Avr n4 or Avr n128 and 3′ TalR Xba+28 and ligated into the *Bam*H1 restriction site of pTAL1 to generate pTALΔ120 and pTALΔ128. The plasmids pTALΔ120 and pTALΔ128 retained the *Esp*3I restriction sites for Golden Gate cloning. TALE arrays cloned into pTALΔ120 and pTALΔ128 were digested with *Bam*H1 and *Xba*I for ligation into pB-Gin-Bam.

To generate mammalian TALER expression vectors, the Gin catalytic domain was PCR amplified from pB-Gin-Avr with 5′ Nhe-SD-Gin F and 3′ GinGS R and ligated into the *Nhe*I and *Bam*HI restriction sites of pcDNA 3.1 (Invitrogen). Avr15 was digested from pTALΔ120 or pTALΔ128 with *Bam*H1 and *Xba*I and ligated into pcDNA-Gin-Bam to generate pcDNA-Gin-Avr expression vectors.

The pBLA substrate plasmids were constructed as previously described (44).

To generate pGL3 reporter plasmids, the SV40 promoter was PCR amplified from pGL3-Promoter (Promega) with the recombination site-containing primers 5′ pGL3 SV40 BglII and 3′ pGL3 SV40 HindIII and ligated into the *Bgl*II and *Hind*III restriction sites of pGL3-Promoter.

### Bacterial recombination assays

Bacterial recombination assays were performed as previously described (44).

### Incremental truncation library

The incremental truncation library was generated using a modified protocol described by Lutz *et al.* (45). Briefly, in order to protect the Gin coding sequence from exonuclease digestion, a stuffer fragment with a *Sma*I restriction site was inserted into *Bam*H1 to generate pB-Gin-SmaI-Bam-Avr. This plasmid was linearized with *Nhe*I and incubated with Exonuclease III for 2.5 min at 37°C followed by heat inactivation at 75°C for 25 min. pB-Gin-Bam-Avr was then incubated with Klenow Fragment (3′–5′ Exo) with 200 µM dNTPs and 5 µM [α]-S-dNTPs for 30 min at 37°C followed by heat inactivation at 80°C for 25 min. To generate the truncation library, pB-Gin-Bam-Avr was incubated with Exonuclease III for 2.5 min at 37°C followed by heat inactivation and subsequent blunt-ending with Mung Bean Nuclease for 1 h at 30°C. After digestion with *Sma*I, the blunt 3′ end of the recombinase coding sequence was ligated to the blunt-ended library of TALE fragments. After transformation and purification, the plasmids were digested with *Sac*I and *Xba*I to release Gin-ΔAvr.

### Mammalian reporter assays

HEK293T cells were seeded onto 96-well plates at a density of 4 × 10^4^ cells per well and grown in a humidified 5% CO_2_ atmosphere at 37°C. At 24 h after seeding, cells were transfected with 150 ng pcDNA TALER expression vector, 2.5 ng pGL3 reporter plasmid, and 1 ng pRL-CMV for expression of *Renilla* luciferase using Lipofectamine 2000 (Invitrogen) according to the manufacturer’s instructions. At 48 h after transfection, cells were lysed with Passive Lysis Buffer (Promega) and luciferase expression was determined using the Dual-Luciferase Reporter Assay System (Promega) according to the manufacturer’s instructions. Luminescence was measured using a Veritas Microplate Luminometer (Turner Biosystems).

## RESULTS

### TALER architecture

**Figure 1.**
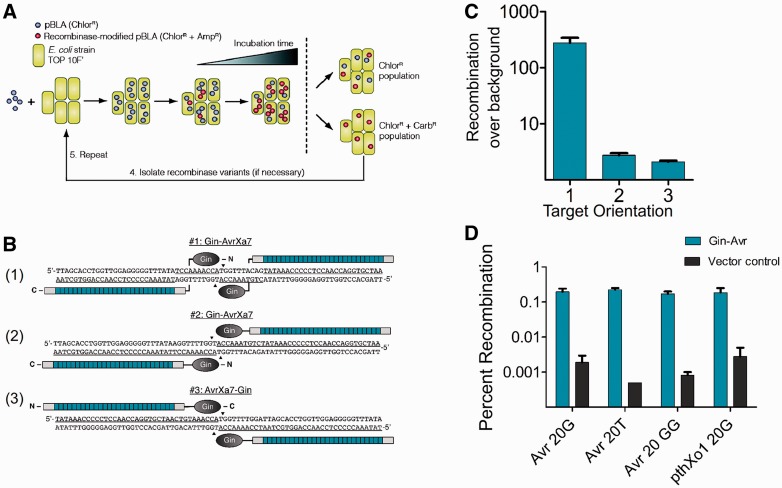
TALER fusion orientation. (**A**) Cartoon illustrating the split β-lactamase system used to evaluate TALER activity. (**B**) Schematic showing the fusion orientation of each TALER and its corresponding target site. (**C**) Activity of each designed TALER fusion against its intended DNA target. Recombination was normalized to background (vector only control). (**D**) Gin-Avr activity against cognate (Avr-20G) and non-cognate (Avr-20T, Avr-20GG, PthXo1-20G) DNA targets. Error bars indicate standard deviation (s.d.) (*n* = 3).

We have recently described a quantitative system for the evaluation and directed evolution of recombinase activity (44). In this system (Figure 1A), a GFPuv transgene flanked by recombination sites is inserted into the gene encoding TEM-1 β-lactamase. This alteration disrupts β-lactamase expression and renders *Escherichia coli* cells that harbor this plasmid (pBLA) susceptible to ampicillin. Expression of an active recombinase from the substrate-containing plasmid, however, leads to recombination between target sites and restoration of the β-lactamase reading frame. This modification establishes host-cell resistance to ampicillin and enables the isolation of active recombinase variants from the substrate-containing plasmid. By measuring the number of ampicillin-resistant transformants following plasmid purification and re-transformation, recombinase activity can be also directly assessed. Because the activity of a chimeric recombinase is dependent upon both the catalytic domain and the DBD, this split gene reassembly selection system can also be used to evaluate the effectiveness of individual DBDs. Thus, we adapted this system to determine an optimal TALER architecture.

Importantly, because the catalytic domain of the DNA invertase Gin and related serine recombinases have pre-defined catalytic specificities, TALER fusion proteins cannot be constructed using the design described for TALENs. Structural and functional studies with the γδ resolvase and designed enzymes have indicated that the C-terminal E-helix mediates serine recombinase DNA recognition (18,46). In ZFRs, this helix binds DNA from the C to the N-terminus, 5′–3′. Thus, because TALEs bind DNA in the 5′–3′ direction, we anticipated that recombination could only occur when the TALE binding site is positioned on the opposite strand of the 20-bp core (Figure 1B).

We choose to generate TALERs using AvrXa7, as this TALE protein has been previously used to generate TALE nucleases and transcription factors (29,30). Conveniently, *Bam*HI restriction sites flank many TALEs, including AvrXa7 and multiple groups have used this restriction site to generate synthetic TALE fusions (29,47). Notably, this *Bam*HI fragment leaves the N-terminus of the TALE intact but removes the native effector domain from the C-terminus. We adopted this strategy and generated a Gin-AvrXa7 fusion by *Bam*H1 restriction digestion.

Gin-AvrXa7 was cloned into a pBLA selection vector containing recombination sites composed of a central 20-bp core sequence, which is recognized by the Gin catalytic domain, and two flanking 26-bp AvrXa7 binding sites. As anticipated, the Gin-AvrXa7 fusion was unable to recombine DNA when AvrXa7 binding sites were positioned adjacent to the 20-bp core (Figure 1C). However, when AvrXa7 binding sites were positioned on the opposite strand of the 20-bp core, recombination was evident (Figure 1C), indicating that recombination site orientation is a critical component for catalytic domain fusion to the TALE N-terminus. In order to further establish that N-terminal fusion is necessary for recombination, we constructed a C-terminal AvrXa7-Gin variant that contained a non-canonical fusion orientation predicted to constrain catalytic domain activity (Figure 1B and Table 1). As expected, we found that this C-terminal AvrXa7 fusion demonstrated negligible activity in bacterial cells (Figure 1C).

**Table 1.** TALER substrates used in study

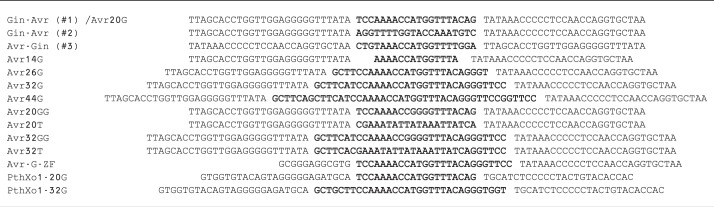

### Designed truncations

**Figure 2.**
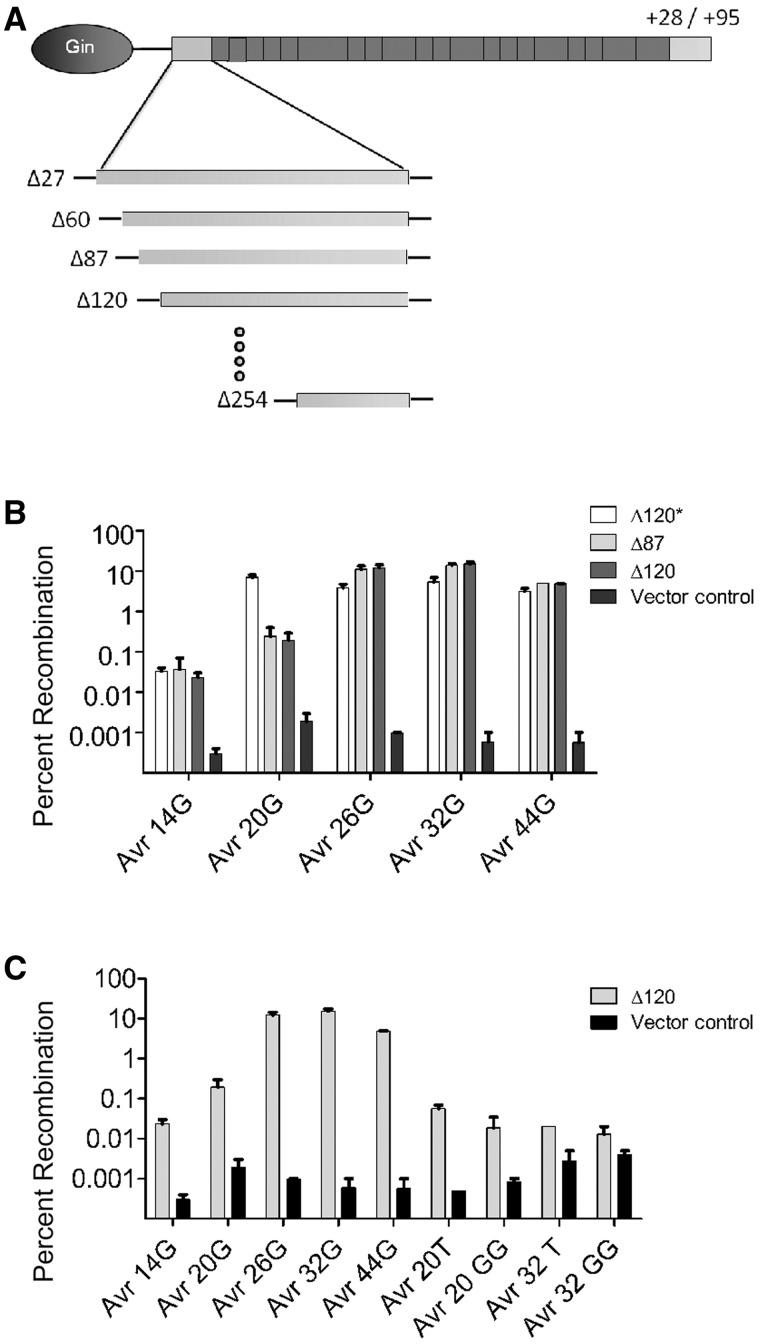
Recombination profiles of selected TALER truncations. (**A**) Schematic illustrating the design of the 20-member TALER truncation library. (**B**) Activity of selected TALER variants against DNA targets containing core sequences of increasing length (14, 20, 26, 32 and 44 bp). (**C**) Gin-AvrXa7Δ120 activity against a diverse panel of substrates containing non-cognate cores sequences or core sites of increasing length. Error bars indicate s.d. (*n* = 3).

Although the Gin-AvrXa7 fusion described above catalysed recombination, the activity of this variant was considerably lower than that of engineered ZFRs (18). Further, specificity analysis revealed that the Gin-AvrXa7 fusion was unable to faithfully discriminate between recognition sites containing non-cognate DBD sites and non-native 20-bp core sequences, indicating that recombination might not be Gin-mediated (Figure 1D). Recent reports have shown that TALEN activity can be enhanced when the TALE portion of the fusion protein is truncated (32,33). Thus, in order to attempt to improve TALER activity, we generated a series of N and C-terminal AvrXa7 truncations (Figure 2A).

Ten N-terminal truncations were assembled at roughly equal intervals beginning at AvrXa7 Thr 27 (Δ27) and ending at AvrXa7 Gly 268 (Δ268) (Supplementary Figure S1). AvrXa7 Δ150, which has been reported as an N-terminal truncation variant for TALENs, was also generated (48). We designed two C-terminal AvrXa7 truncations at positions 28 (+28) and 95 (+95). Both +28 and +95 have been reported as stable fusion points in TALENs (32). Each TALE truncation variant was fused to the Gin catalytic domain and this 20-member TALER library was cloned into a pBLA selection vector containing Avr-20G recognition sites. Following one round of selection in bacterial cells (see ‘Materials and Methods’ section), we sequenced individual ampicillin-resistant clones and found that all selected TALERs contained either one of two N-terminal truncations: Δ87 and Δ120. Each selected clone was also +28 on the C-terminus. With the exception of a single Δ120 clone with a spontaneous 12 amino acid deletion near the fusion point (Δ120*), the activity of these clones was quite low (Figure 2B). In this assay, Gin-based ZFRs routinely show 20–40% recombination, however, the highest activity observed among the selected TALER fusions was ∼7% recombination (Gin-AvrXa7Δ120*). Because the TALE DBD is three times larger than a ZF domain (not including the required flanking peptide sequence), we reasoned that the 20-bp spacer used for these TALER constructs might not be the optimal length for recombination.

### Core sequence length

We next investigated the effect core sequence length has on recombination by evaluating whether DNA targets containing 14 (Avr-14G), 26 (Avr-26G) and 32-bp (Avr-32G) core sites could be recombined by selected TALERs. In order to maintain the reading frame of the β-lactamase gene following recombinase-mediated reassembly, we modified core half-sites by ± 3 bp (Table 1). We subjected the 20-member TALER library described above to one round of selection against each target site variant. Although we were unable to identify TALER variants capable of recombining the shortest target, Avr-14G (data not shown), we identified two Gin-ΔAvrXa7 variants (based on the N-terminal TALE truncations Δ87 and Δ120 and the C-terminal truncation +28) that recombined Avr-26G and Avr-32G. In particular, clonal analysis revealed that the selected TALERs (Gin-AvrXa7Δ87 and Gin-AvrXa7Δ120) recombined DNA with longer cores (e.g. 26 and 32 bp) at least 100-fold more efficiently than shorter cores (e.g. 14 and 20 bp) (Figure 2B). Further, we found that Gin-AvrXa7Δ120 recombined targets containing a cognate core sequence (Avr-26G and Avr-32G) >100-fold more efficiently than a non-cognate core (Avr-20T, Avr-20GG, Avr-32T and Avr-32GG) (Figure 2C). Interestingly, the Gin-AvrXa7Δ120 fusion was not as active on 44-bp cores (Avr-44G) (recombination was ∼3-fold lower than Avr-32G) (Figure 2C), indicating that core lengths between 26 and 44 bp are likely optimal for recombination by Gin-AvrXa7Δ120 in *E*. *coli*.

### Incremental truncation library

Although Gin-AvrXa7Δ120 showed increased recombination in comparison to Gin-AvrXa7, we suspected that Gin-AvrXa7Δ120 might not be an optimal TALE fusion architecture because: (i) ZFRs containing the Gin catalytic domain recombined DNA >2-fold more efficiently than Gin-AvrXa7Δ120 and (ii) Gin-AvrXa7Δ120 was not identified from a comprehensive library of TALE truncation variants. Thus, in order to identify better fusion architectures, we devised a screen based on the generation of a library of incrementally truncated TALE DBDs.

To achieve this, we adapted a protocol described by Lutz *et al.* (45) to enable fusion of an unmodified N-terminal domain (Gin) to a library of truncated C-terminal fragments (AvrXa7) (see Materials and Methods section). N-terminal AvrXa7 truncations that spanned the region between the AvrXa7 N-terminus (Met 1) and the first AvrXa7 repeat (Leu 298) were generated by exonuclease digestion and fused to an unmodified copy of the Gin catalytic domain (theoretical number of protein variants: ∼300). Because previous results indicated that +28 is the optimal C-terminal truncation, we incorporated this architecture into the truncation library. TALERs were cloned into a pBLA selection vector containing Avr-32G target sites and transformed into *E. coli* (>1 × 10^5^ transformants). Sequence analysis confirmed an equal distribution of truncations spanning the region of interest (data not shown).

**Figure 3.**
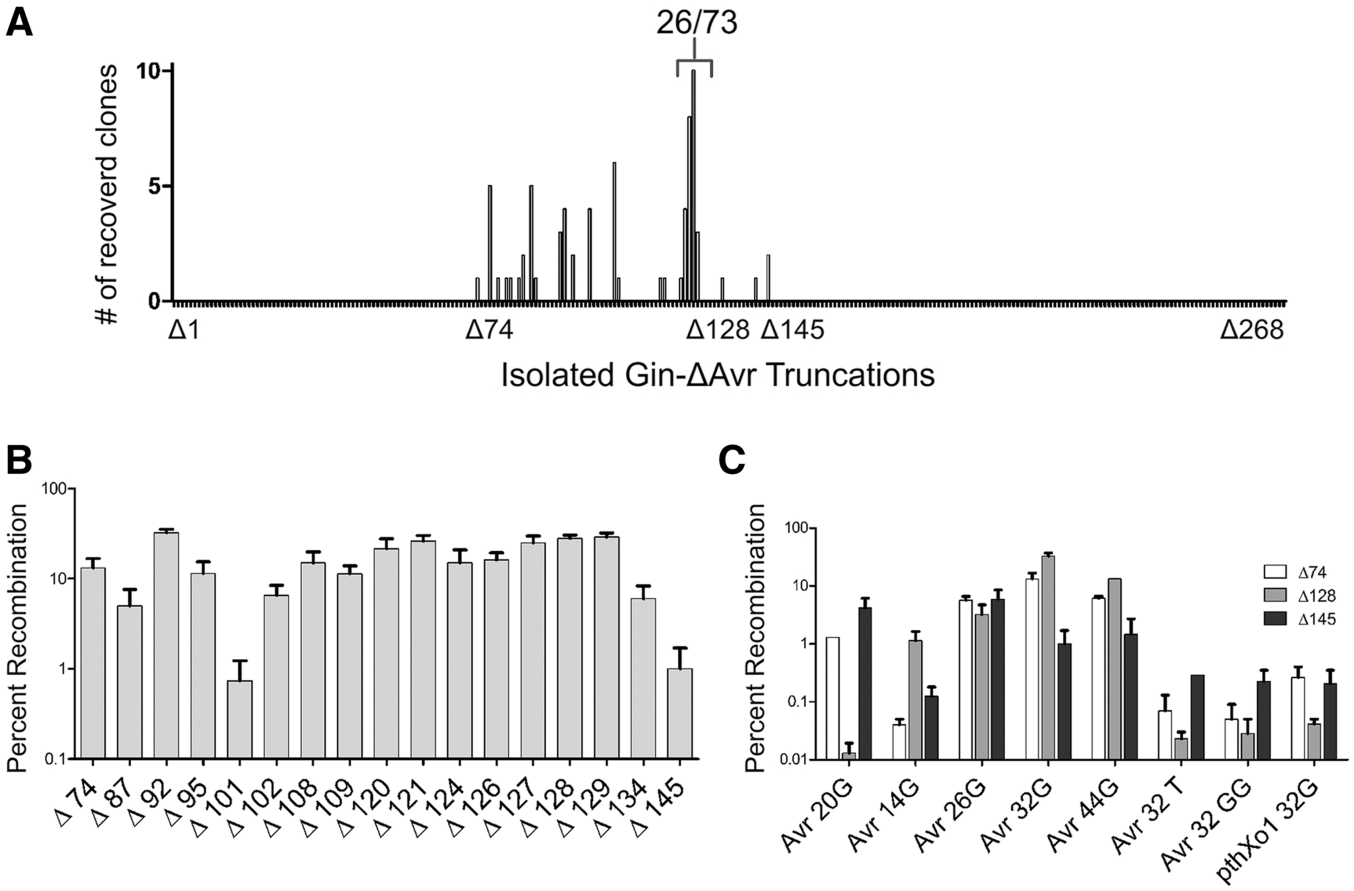
TALER variants selected from incremental truncation library. (**A**) Frequency of selected TALER truncation variants. After three rounds of selection, incrementally truncated Gin-AvrXa7 variants were isolated and DNA sequencing was used to determine truncation length. (**B**) Activity of incrementally truncated TALER variants (between Δ92 and Δ134 in length) against the Avr-32G DNA target. For reference, the shortest (Δ145) and longest (Δ74) truncation variants, as well as Δ87 were included. (**C**) Activity of Gin-AvrΔ74, Gin-AvrΔ128 and Gin-AvrΔ145 against a diverse panel of cognate and non-cognate DNA targets. Error bars indicate s.d. (*n* = 3).

Following three rounds of selection, we sequenced individual ampicillin-resistant clones and identified a number of unique truncation variants (Figure 3A). Consistent with the selections performed using the 20-member TALE truncation library, which suggested that the optimal N-terminal TALER fusion points were likely located in proximity to positions 87 and 120, all selected Gin-AvrXa7 variants were found to contain a truncation between positions 74 (Δ74) and 147 (Δ147). In particular, 26 of 73 (35.6%, *P* < 0.001) clones contained truncations between positions 124 (Δ124) and 129 (Δ129). From this population, truncations at position 128 (Δ128) were among the most represented.

In order to systematically determine whether selected AvrXa7 domains increased TALER activity, we evaluated the performance of isolated Gin-AvrXa7 variants against DNA substrates containing Avr-32G target sites in *E. coli*. We focused our analysis on clones containing N-terminal deletions between AvrXa7 position 92 (Δ92) and 134 (Δ134). Consistent with sequence analysis, we found that TALERs containing N-terminal truncations between Δ120 and Δ129 recombined DNA more efficiently than variants based on comparatively longer or shorter truncations, although the Δ92 fusion was also quite active (Figure 3B). We then characterized three clones further: Δ74 and Δ145 were chosen because they represented the boundaries of possible fusion points, and Δ128 was assayed because it was the most prevalent clone found in the selections. Five targets with spacer lengths from 14 to 44-bp were assayed along with three negative controls (Avr32T, Avr32GG and PthXo1-32G). We found that Gin-Avr32GΔ74 and Gin-Avr32GΔ145 had modest activity on spacers longer than 20 bp, whereas Gin-Avr32GΔ128 recombined DNA with efficiencies comparable to the ZFR GinC4 (Figure 3C). Furthermore, specificity analysis revealed that Gin-Avr32GΔ74, Gin-Avr32GΔ128 and Gin-Avr32GΔ145 could recombine substrates harboring cognate cores >100-fold more efficiently than non-cognate cores (Avr-32T, Avr-32GG and PthXo1-32G) (Figure 3C). Together, these results suggest that TALE proteins containing N-terminal deletions between Δ120 and Δ129 represent an optimal truncation for fusion to a recombinase.

### Incorporation of synthetic TALE repeat arrays

The studies described above used the native DBDs of the naturally occurring AvrXa7 TALE protein. In order to determine whether designed TALE repeat arrays can be incorporated into the selected Gin-ΔAvrXa7 frameworks, we generated a series of synthetic TALE proteins (15–20 repeats in length) designed to target the AvrXa7 binding site (Supplementary Figure S2). TALE proteins were constructed using a publicly available TALEN plasmid set (Addgene) (27). The cloning plasmid was modified to include the +28 C-terminal truncation and either the Δ120 or Δ128 N-terminal truncation. Designed TALEs were fused to the Gin catalytic domain (denoted as Gin-Avr15Δ120 and Gin-Avr15Δ128) and cloned into a pBLA selection vector containing Avr-32G or Avr-32T target sites.

**Figure 4.**
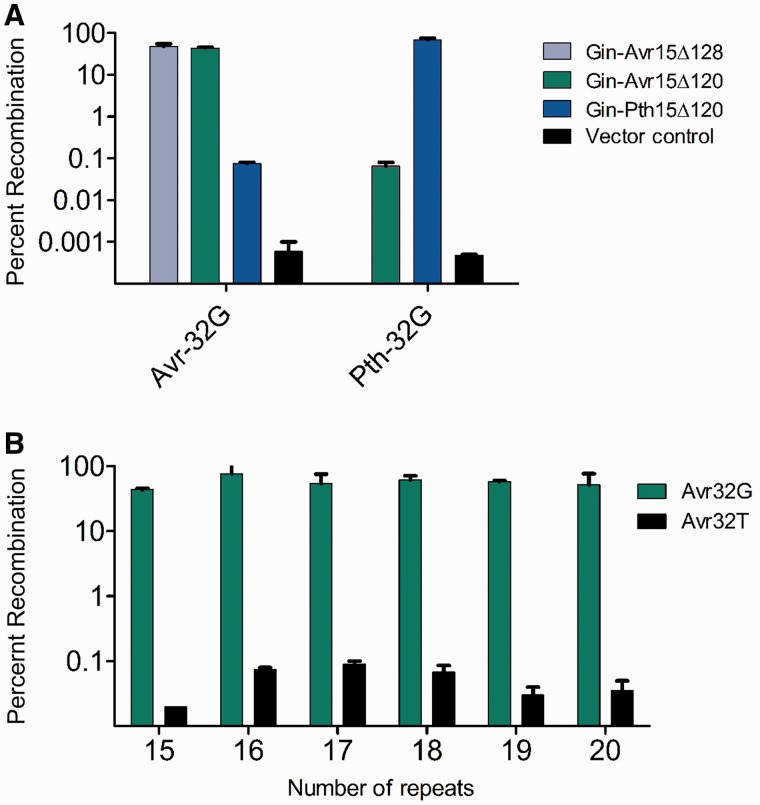
Activity of synthetic TALERs. (**A**) Activity of synthetic Gin-Avr15Δ128, Gin-Avr15Δ120 and Gin-Pth15Δ120 variants against the DNA targets Avr-32G or Pth-32G. (**B**) Activity of synthetic TALERs with DBDs between 15 and 20 repeats in length based on Gin-AvrΔ120 against Avr-32G and Avr-32T. Error bars indicate s.d. (*n* = 3).

Activity analysis in *E. coli* revealed that both Gin-Avr15Δ120 and Gin-Avr15Δ128 could be used to recombine DNA when fused to an active catalytic domain and that incorporation of synthetic repeats provided an increase in activity (Figure 4A). Importantly, each TALER displayed stringent selectivity, recombining target sites that contained cognate cores >1000-fold more efficiently than non-cognate cores (Figure 4B). Surprisingly, TALERs based on the Δ120 truncation were also found to recombine DNA as effectively as TALEs based on the Δ128 architecture (Figure 4A), indicating that designed TALEs may be less sensitive to N-terminal truncation than those containing the native AvrXa7 DBD.

To further demonstrate that the TALER architecture described herein can be reprogrammed to target any DNA sequence, we created a synthetic enzyme designed to target the sequence recognized by the naturally occurring TALE protein PthXo1 (Gin-Pth15Δ120). We found that Gin-Pth15Δ120 was highly active on its cognate substrate and that both Gin-Pth15Δ120 and Gin-Avr15Δ120 showed a >600-fold increase in recombination for targets with their cognate binding sites (Figure 4A). We also assessed the activity of a series of designed TALERs containing DBDs between 15 and 20 repeats in length and found that each fusion catalysed recombination with similarly high efficiency and specificity (Figure 4B), demonstrating that chimeric recombinases that incorporate synthetic TALE repeat arrays can be used for site-specific recombination.

### TALER activity in mammalian cells

We next set out to determine whether TALERs could modify DNA in mammalian cells. To achieve this, we used an episomal reporter assay that enables rapid assessment of recombinase activity in cell culture. In this assay, human embryonic kidney (HEK) 293T cells are co-transfected with a recombinase expression vector and a reporter plasmid (pGL3) that contains a luciferase gene under the control of a SV40 promoter flanked by recombination sites. Transient expression of the appropriate recombinase leads to excision of the SV40 promoter and reduced luciferase expression in cells. Recombinase activity is thus directly proportional to the fold-reduction in luciferase expression.

**Figure 5.**
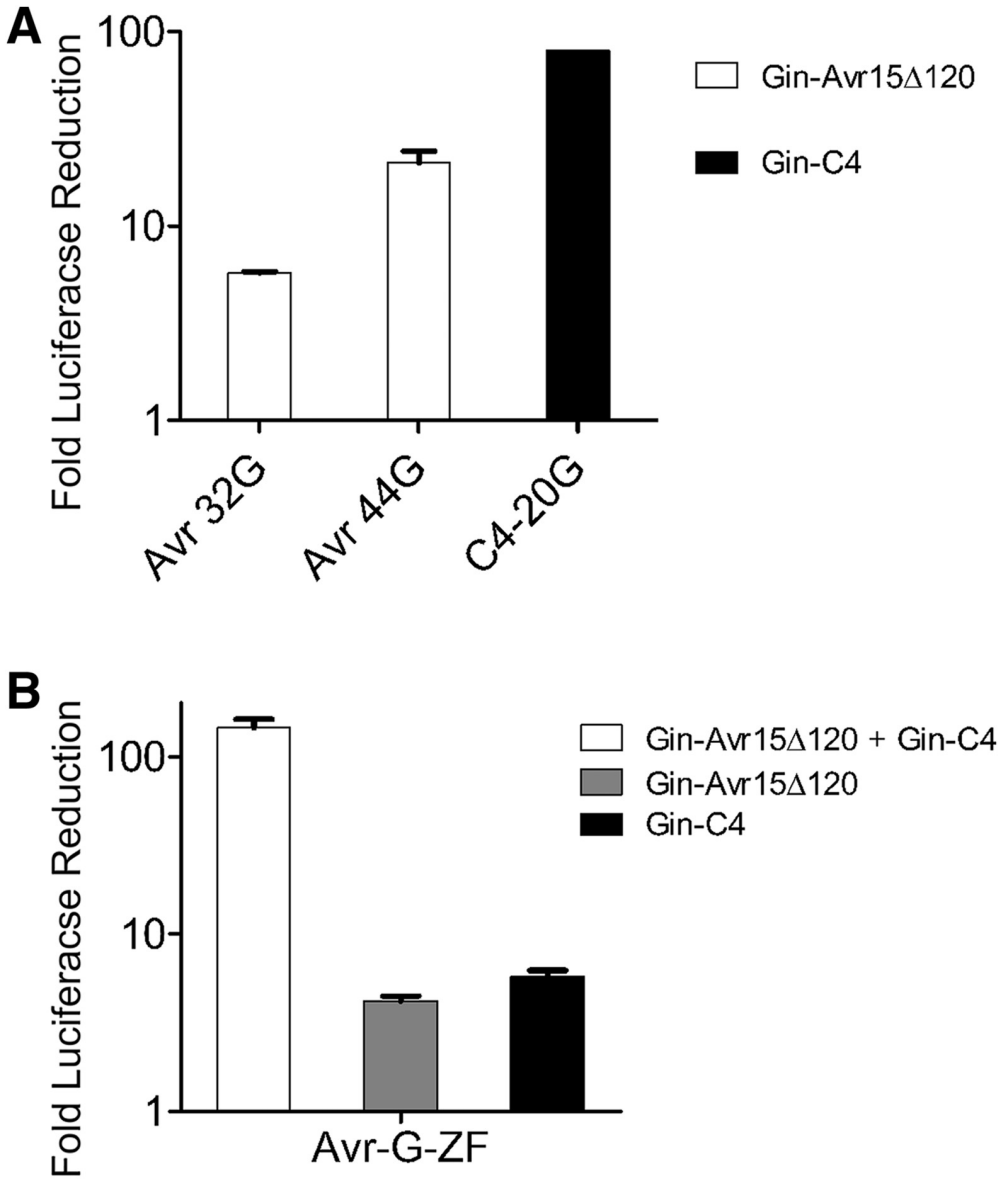
TALER activity in mammalian cells. (**A**, **B**) Fold-reduction of luciferase expression in HEK293T cells co-transfected with (A) TALER or ZFR expression vectors (Gin-AvrΔ120 and GinC4) in the presence of reporter plasmid (Avr-32G, Avr-44G and C4-20G) or (B) TALER and ZFR expression vector in combination (Gin-AvrΔ120 + GinC4) with reporter plasmid (Avr-G-ZF). Error bars indicate s.d. (*n* = 3).

Co-transfection of Gin-Avr15Δ120 with a reporter plasmid harboring Avr-44G recognition sites (pGL3-Avr-44G) led to a ∼20-fold reduction in luciferase expression as compared to transfection of pGL3-Avr-44G alone (Figure 5A). Despite the fact that Gin-Avr15Δ120 showed similar activity to the ZFR GinC4 in *E. coli*, we found that GinC4-reduced luciferase expression by >80-fold after co-transfection with its cognate target plasmid, pGL3-C4-20G (Figure 5A). We believe this discrepancy may be due to the comparatively shorter intervening DNA sequence between recombinase target sites in pGL3 than pBLA or differential expression between TALERs and ZFRs in mammalian cells. The underlying cause for this disparity, however, remains unclear. Finally, although 32 bp was determined to be the optimal core sequence length for TALERs in *E. coli*, we found that co-transfection of Gin-Avr15Δ120 with pGL3-Avr-32G led to only a 6-fold reduction in luciferase expression (Figure 5A). The underlying cause behind this disparity also remains unclear.

We next investigated whether a ZFR (GinC4) and a TALER (Gin-Avr15Δ120) could form a compatible heterodimer in mammalian cells. To evaluate this possibility, we generated a hybrid recombination site in which the AvrXa7 binding site and the C4 zinc-finger binding site (GCG GGA GGC GTG) flank the core sequence recognized by the Gin catalytic domain (pGL3-Avr-G-ZF) (Table 1). Surprisingly, co-transfection of pGL3-Avr-G-ZF with GinC4 and Gin-Avr15Δ120 led to a >140-fold reduction in luciferase expression as compared to pGL3-Avr-G-ZF alone (Figure 5B), whereas transfection with either GinC4 or Gin-Avr15Δ120 with pGL3-Avr-G-ZF led to a negligible decrease in reporter gene expression. These results demonstrate that generating ZF-TALE heterodimers represents a potentially effective approach for improving the targeting capacity of chimeric recombinases.

## DISCUSSION

Unlike ZFPs, which contain a very minimal fusion architecture, TALE DBDs require native protein framework on either side of the DBD array to function. The so-called 0th and 1st repeats, which mediate binding of the thymine residue at position 0 and are found in almost all known TALE recognition sites, represent such an N-terminal framework. A recent crystal structure provided a description of the binding of the position 0 thymine, yet there remains insufficient data to determine a minimal TALE architecture (45). Indeed, all studies to date have used an N-terminal truncation containing considerably more residues than those required to mediate binding at position 0. It remains uncertain what role this part of the protein has in enabling the proper DNA binding conformation or what might constitute a minimal TALE domain. Although initial attempts to generate functional TALE chimeras were based on fusion to full-length TALE proteins, more recent studies have focused on the identification of unique C-terminal truncations that improve effector domain function in the context of the Δ150 N-terminal architecture (32,33,36,47,49). A previous report indicated that deletion of N-terminal residues 2-153 (Δ150) of the AvrBs3 TALE removes the domain required for translocation of the TALE from its native bacteria to the target plant cell but does not compromise transcription factor activity (48).

Developing an active TALER, however, necessitated that unique N-terminal TALE variants be identified. We initially conducted a broad, systematic survey of N-terminal TALEs with the C-terminal truncations +28 and +95 and found that only two domains (Δ87 with +28 and Δ120 with +28) demonstrated sufficiently high activity for further analysis. A secondary analysis based on incremental truncation of the AvrXa7 N-terminus led to the identification of a broad cluster of truncation variants centered between AvrXa7 position 74 (Δ74) and position 145 (Δ145). Of the clones recovered in this experiment, 38% contained truncations between positions Δ119 and Δ128, and a survey of data obtained on TALERs with fusions in this region showed high activity. In particular, we found that TALERs based on N-terminal truncations from this region (Δ128 and Δ120) could be used to recombine DNA in bacteria and mammalian cells. The clustering of truncation variants between Δ119 and Δ128 may also be indicative of the intrinsic stability of this region.

ZFRs typically catalyse recombination between target sites 44 and 50 bp in length. Each target site contains a central 20-bp core sequence, which is recognized by the recombinase catalytic domain, and two adjacent ZFP binding sites. The fusion orientation of TALERs, however, necessitates that TALE binding sites are on the opposite strand relative to the central core sequence. This unique geometry led us to investigate the minimum core sequence requirements for recombination. Because of the length of TALE DBDs (TALE repeats are 3–4 times longer than ZFPs) and the extended N-terminal linker between the catalytic domain and the TALE domain, we reasoned that longer core sequences (32 or 44 bp) would be necessary for recombination. Indeed, with the exception of a TALE variant harboring a spontaneous deletion (Δ120*), most N-terminal truncation variants identified in this study demonstrated optimal performance against 32-bp cores. These results are consistent with those reported with TALENs, which unlike ZFNs require significantly longer spacer sequences (e.g. TALENs: 17–20 bp, ZFNs: 5–6 bp) to efficiently cleave DNA (32). In support of these observations, we found that selection for unique N-terminal truncation variants against a short core sequence (14 bp) did not yield any clones.

Gin-AvrXa7Δ128 was identified as an optimal TALE fusion, but subsequent studies using synthetic TALE proteins generated using a publicly available TALE assembly kit indicated that Δ128 and Δ120-based TALERs showed similar activity in *E. coli*. These designed TALEs were based on a chimeric protein derived from the closely related and naturally occurring Tal1c and PthXo1 TALE proteins. Although TALEs share high homology, they are not identical. While polymorphisms in RVD repeats outside of residues 12 and 13 have been shown to have no affect on TALE fusion activities, to our knowledge no systematic evaluation of differences in TALE framework outside the DBDs has been reported (50). As demonstrated by the analysis of the incremental truncation library, minor amino acid alterations can significantly influence the activity of a particular fusion. Thus, some of the discrepancy in activity we observed between Gin-AvrXa7Δ120 and the synthetic Gin-Avr15Δ120 may be attributable to the sequence variations between AvrXa7 framework and the TALE framework architecture used by Cermak *et al.* (27).

The four RVDs (NI: A, HD: C, NG: T, and NN: G) favored for construction of synthetic TALEs are the most prevalent in nature; however, it remains to be determined whether these repeats represent the most specific RVD modules. For the 26-repeat AvrXa7 TALE, a synthetic version targeting the same sequence would have 16 changes in RVD composition (Supplementary Figure S2). We hypothesize that because they are more commonly found in nature, the four RVDs selected for synthetic use might have a higher affinity for their cognate bases than other RVDs. If this were the case, it would be reasonable to assume that a TALE created with the synthetic RVD repeats could have higher DNA-binding affinity than a TALE using the native domains. Although we did not directly address the issue of RVD affinity, we did find that TALERs containing synthetic repeat arrays were more active than constructs, which contained the native AvrXa7 DBD. TALERs with synthetic DBDs showed ∼2-fold higher activities than constructs containing the native repeats, despite containing significantly fewer DBDs. Additionally, the gain in activity observed with the synthetic arrays was not correlated with any increase in off-target recombination.

Several studies have shown that TALEs can tolerate some mismatches in their target sequence (49,51,52). These findings are unsurprising, as RVDs that are positively associated with particular bases have been shown to tolerate non-cognate bases in nature. The cooperative specificity afforded by TALERs could be used to circumvent potential limitations, however. Because the catalytic domain contributes specificity to recombination, we envision that designer TALERs capable of selectively modifying highly homologous genomic sequences could be generated as well. Indeed, our laboratory has recently demonstrated that recombinase catalytic specificity can be effectively reprogrammed to target unnatural core sites (18). Our results from TALER optimization were not completely portable to our mammalian assay. Whether this incongruity is the result of features inherent to the mammalian cell assay, or an indication that TALERs will require further optimization for increased activity in mammalian cells is currently under investigation. Regardless, we believe that the refined TALER architecture presented will serve as an ideal starting point for future work. By combining our ongoing efforts in creating novel recombinase domains with improvements in the TALER architecture described herein, genomic modification with chimeric recombinases may soon become a routine enterprise.

## SUPPLEMENTARY DATA

Supplementary Data are available at NAR Online: Supplementary Table 1, Supplementary Figures 1–2 and Supplementary Datasets.

## FUNDING

National Institute of Health (NIH) [DP1CA174426]; Skaggs Institute for Chemical Biology; National Institute of General Medicine Sciences Training Grant [T32GM080209 to T.G.]. Funding for open access charge: Grants and internal funding.

*Conflict of interest statement*. Patent application covering this work has been filed.

## Supplementary Material

Supplementary Data
