# Supplementary material for: Chimeric TALE recombinases with programmable DNA sequence specificity
Source: Nucleic Acids Res. 2012 Sep 26;40(21):11163–72. doi: 10.1093/nar/gks875 (PMC3510496; doi:10.1093/nar/gks875)
Supplement: Supplementary Data [file supp_40_21_11163__index.html]

Chimeric TALE recombinases with programmable DNA sequence specificity — Chimeric TALE recombinases with programmable DNA sequence specificity — Supplementary Data 

# Chimeric TALE recombinases with programmable DNA sequence specificity

## Supplementary Data

files

**Files in this Data Supplement:**

- Supplementary Data - docx file
- Supplementary Data - xlsx file
