# Supplementary material for: Chimeric TALE recombinases with programmable DNA sequence specificity
Source: Nucleic Acids Res. 2012 Sep 26;40(21):11163–72. doi: 10.1093/nar/gks875 (PMC3510496; doi:10.1093/nar/gks875)
Supplement: Supplementary Data [file supp_gks875_nar-01819-h-2012-File007.docx]

**Supplementary Information for:**

Chimeric TALE recombinases with programmable DNA binding specificity.

Andrew C. Mercer^1^, Thomas Gaj^1^, Roberta P. Fuller^1^, and Carlos F. Barbas III.^1,*^

**Supplementary Figure 1**. Location of primers for N-terminal designed truncations. Red star denotes the location of Δ120 fusion point.

**Supplementary Figure 2**. Comparison of native and synthetic RDV domains for the AvrXa7 target sequence.

**TALE and TALER sequences**

**AvrXa7 protein**:

MDPIRSRTPSPARELLPGPQPDRVQPTADRGGAPPAGGPLDGLPARRTMSRTRLPSPPAPSPAFSAGSFSDLLRQFDPSLLDTSLLDSMPAVGTPHTAAAPAECDEVQSGLRAADDPPPTVRVAVTAARPPRAKPAPRRRAAQPSDASPAAQVDLRTLGYSQQQQEKIKPKVRSTVAQHHEALVGHGFTHAHIVALSQHPAALGTVAVTYQDIIRALPEATHEDIVGVGKQWSGARALEALLTEAGELRGPPLQLDTGQLLKIAKRGGVTAVEAVHAWRNALTGAPLNLTPDQVVAIASNIGGKQALETVQRLLPVLCQDHGLTPDQVVAIASHGGGKQALETVQRLLPVLCQDHGLTPDQVVAIASNIGGKQALETVQRLLPVLCQAHGLTPDQVVAIASNIGGKQALETVQRLLPVLCQDHGLTPAQVVAIASNSGGKQALETVQRLLPVLCQDHGLTPDQVVAIASHDGGKQALETLQRLLPVLCQDHGLTPDQVVAIANNNGGKQALETLQRLLPVLCQDHGLTPDQVVAIASHDGGKQALETVQRLLPVLCQDHGLTPDQVVAIASHDGGKQALETVQRLLPVLCQDHGLTPAQVVAIASHDGGKQALETVQRLLPVLCQDHGLTPDQVVAIASNSGGKQALETVQRLLPVLCQDHGLTPDQVVAIASNGGKQALETVQRLLPVLCQDHGLTPDQVVAIASNGGKQALETVQRLLPVQRLLPVLCQDHGLTQDQVVAIASHDGGKQALETVQRLLPVLCQDHGLTPDQVVAIASHDGGKQALETVQRLLPVLCQDHGLTPDQVVAIASNSGGKQALETVQRLLPVLCQDHGLTPDQVVAIASNSGGKQALETVQRLLPVLCQDHGLTPDQVVAIASNNGGKQALETVQRLLPVLCQDHGLTPDQVVAIANNNGGKQALETVQRLLPVLCQDHGLTPAQVVAIASNIGGKQALETVQRLLPVLCQDHGLTLDQVVAIASNGGSKQALETVQRLLPVLCQDHGLTPDQVVAIANNNGGKQALETVQRLLPVLCQDHGLTPDQVVAIASNIGGKQALETVQRLLPVLCQDHGLTLDQVVAIASNGGKQALETVQRLLPVLCQDHGLTPNQVVAIASNSGGKQALETVQRLLPVLCQDHGLTPNQVVAIASNGGKQALESIVAQLSRPDPALAALTNDHLVALACLGGRPALDAVKKGLPHAPELIRRINRRIPERTSHRVPDLAHVVRVLGFFQSHSHPAQAFDDAMTQFEMSRHGLVQLFRRVGVTEFEARYGTLPPASQRWDRILQASGMKRAKPSPTSAQTPDQASLHAFADSLERDLDAPSPMHEGDQTRASSRKRSRSDRAVTGPSTQQSFEVRVPEQQDALHLPLSWRVKRPRTRIGGGLPDPGTPIAADLAASSTVMWEQDAAPFAGAADDFPAFNEEELAWLMELLPQSGSVGGTI

**AvrXa7 gene**:

ATGGATCCCATTCGTTCGCGCACGCCAAGTCCTGCCCGCGAGCTTCTGCCCGGACCCCAACCGGATAGGGTTCAGCCGACTGCAGATCGGGGGGGGGCTCCGCCTGCTGGCGGCCCCCTGGATGGCTTGCCCGCTCGGCGGACGATGTCCCGGACCCGGCTGCCATCTCCCCCTGCGCCCTCGCCTGCGTTCTCGGCGGGCAGCTTCAGCGATCTGCTCCGTCAGTTCGATCCGTCGCTTCTTGATACATCGCTTCTTGATTCGATGCCTGCCGTCGGCACGCCGCATACAGCGGCTGCCCCAGCAGAGTGCGATGAGGTGCAATCGGGTCTGCGTGCAGCCGATGACCCGCCACCCACCGTGCGTGTCGCTGTCACTgcgGCGCGGCCGCCGCGCGCCAAGCCGGCCCCGCGACGGCGTGCGGCGCAACCCTCCGACGCTTCGCCGGCCGCGCAGGTGGATCTACGCACGCTCGGCTACAGTCAGCAGCAGCAAGAGAAGATCAAACCGAAGGTGCGTTCGACAGTGGCGCAGCACCACGAGGCACTGGTGGGCCATGGGTTTACACACGCGCACATCGTTGCGCTCAGCCAACACCCGGCAGCGTTAGGGACCGTTGCTGTCACGTATCAGGACATAATCAGGGCGTTGCCAGAGGCGACACACGAAGACATCGTTGGCGTCGGCAAACAGTGGTCCGGCGCACGCGCTCTGGAGGCCTTGCTCACGGAGGCGGGGGAGTTGAGAGGTCCGCCGTTACAGTTGGACACAGGCCAACTTCTCAAGATTGCAAAACGTGGCGGCGTGACCGCAGTGGAGGCAGTGCATGCATGGCGCAATGCACTGACGGGTGCCCCCCTGAACCTGACCCCGGACCAAGTGGTGGCCATCGCCAGCAATATTGGCGGCAAGCAGGCGCTGGAGACGGTACAGCGGCTGTTGCCGGTGCTGTGCCAGGACCATGGCCTGACCCCGGACCAGGTCGTGGCCATCGCCAGCCATGGCGGCGGCAAGCAGGCGCTGGAGACGGTGCAGCGGCTGTTGCCGGTGCTGTGCCAGGACCATGGCCTGACCCCGGACCAGGTGGTGGCCATCGCCAGCAATATTGGCGGCAAGCAGGCGCTAGAGACGGTGCAGCGGCTGTTGCCGGTGCTGTGCCAGGCCCATGGCCTGACCCCGGACCAGGTCGTGGCCATCGCCAGCAATATTGGCGGCAAGCAGGCGCTGGAGACGGTGCAGCGGCTGTTGCCGGTGCTGTGCCAGGACCATGGCCTGACCCCGGCCCAGGTGGTGGCCATCGCCAGCAATAGTGGCGGCAAGCAGGCGCTGGAGACGGTGCAGCGGCTGTTGCCGGTGCTGTGCCAGGACCATGGCCTGACCCCGGACCAAGTCGTGGCCATCGCCAGCCACGATGGCGGCAAGCAGGCGCTGGAGACGCTGCAGCGGCTGTTGCCGGTGCTGTGCCAGGACCATGGCCTGACCCCGGACCAGGTCGTGGCCATCGCCAACAATAACGGCGGCAAGCAGGCGCTGGAGACGCTGCAGCGGCTGTTGCCGGTGCTGTGCCAGGACCATGGCCTGACCCCGGACCAAGTGGTGGCCATCGCCAGCCACGATGGCGGCAAGCAGGCGCTGGAGACGGTGCAGCGGCTGTTGCCGGTGCTGTGCCAGGACCATGGCCTGACCCCGGACCAGGTGGTGGCCATCGCCAGCCACGATGGCGGCAAGCAGGCGCTGGAGACGGTGCAGCGGCTGTTGCCGGTGCTGTGCCAGGACCATGGCCTGACCCCGGCCCAAGTGGTGGCCATCGCCAGCCACGATGGCGGCAAGCAGGCGCTGGAGACGGTGCAGCGGCTGTTGCCGGTGCTGTGCCAGGACCATGGCCTGACCCCGGACCAGGTGGTGGCCATCGCCAGCAATAGCGGCGGCAAGCAGGCGCTGGAGACGGTACAGCGGCTGTTGCCGGTGCTGTGCCAGGACCATGGACTGACCCCGGACCAGGTCGTGGCCATCGCCAGCAATGGCGGCAAGCAGGCGCTGGAGACGGTACAGCGGCTGTTGCCGGTGCTGTGCCAGGACCATGGCCTGACCCCGGACCAGGTCGTGGCCATCGCCAGCAATGGCGGCAAGCAGGCGCTGGAGACGGTGCAGCGGCTGTTGCCGGTACAGCGGCTGTTGCCGGTGCTGTGCCAGGACCATGGCCTGACCCAGGACCAGGTGGTGGCCATCGCCAGCCACGATGGCGGCAAGCAGGCGCTGGAGACGGTGCAGCGGCTGTTGCCGGTGCTGTGCCAGGACCATGGCCTGACCCCGGACCAAGTGGTGGCCATCGCCAGCCACGATGGCGGCAAACAGGCGCTGGAGACGGTGCAGCGGCTGTTGCCGGTGCTGTGCCAGGACCATGGCCTGACCCCGGACCAGGTGGTGGCCATCGCCAGCAATAGTGGCGGCAAGCAGGCGCTGGAGACGGTGCAGCGGCTGTTGCCGGTGCTGTGCCAGGACCATGGCCTGACCCCGGACCAAGTGGTGGCCATCGCCAGCAATAGTGGCGGCAAGCAGGCGCTGGAGACGGTGCAGCGGCTGTTGCCGGTGCTGTGCCAGGACCATGGCCTGACCCCGGACCAGGTGGTGGCCATCGCCAGCAATAACGGCGGCAAGCAGGCGCTGGAGACGGTGCAGCGGCTGTTGCCGGTGCTGTGCCAGGACCATGGCCTGACCCCGGACCAGGTCGTGGCCATCGCCAACAATAACGGCGGCAAGCAGGCGCTGGAGACGGTGCAGCGGCTGTTGCCGGTGCTGTGCCAGGACCATGGCCTGACCCCGGCGCAGGTGGTGGCCATCGCCAGCAATATTGGCGGCAAGCAGGCGCTGGAGACGGTGCAGCGGCTGTTGCCGGTGCTGTGCCAGGACCATGGCCTGACCCTGGACCAGGTGGTGGCCATTGCCAGCAATGGCGGCAGCAAACAGGCGCTAGAGACGGTGCAGCGGCTGTTGCCGGTGCTGTGCCAGGACCATGGCCTGACCCCGGACCAAGTGGTGGCCATCGCCAACAATAACGGCGGCAAGCAGGCGCTGGAGACGGTGCAGCGGCTGTTGCCGGTGCTGTGCCAGGACCATGGCCTGACCCCGGACCAGGTCGTGGCCATCGCCAGCAATATTGGCGGCAAGCAGGCGCTGGAGACGGTGCAGCGGCTGTTGCCGGTGCTGTGCCAGGACCATGGCCTGACCCTGGACCAGGTGGTGGCCATCGCCAGCAATGGCGGCAAGCAGGCGCTGGAGACGGTGCAGCGGCTGTTGCCGGTGCTGTGCCAGGACCATGGCCTGACCCCGAACCAGGTGGTGGCCATCGCCAGCAATAGTGGCGGCAAGCAGGCGCTGGAGACGGTGCAGCGGCTGTTGCCGGTGCTGTGCCAGGACCATGGCCTGACCCCGAACCAGGTGGTGGCCATCGCCAGCAATGGCGGCAAGCAGGCGCTGGAGAGCATTGTTGCCCAGTTATCTCGCCCTGATCCGGCGTTGGCCGCGTTGACCAACGACCACCTCGTCGCCTTGGCCTGCCTCGGCGGACGTCCTGCCCTGGATGCAGTGAAAAAGGGATTGCCGCACGCGCCGGAATTGATCAGAAGAATCAATCGCCGCATTCCCGAACGCACGTCCCATCGCGTTCCCGACCTCGCGCACGTGGTTCGCGTGCTTGGTTTTTTCCAGAGCCACTCCCACCCAGCGCAAGCATTCGATGACGCCATGACGCAGTTCGAGATGAGCAGGCACGGCTTGGTACAGCTCTTTCGCAGAGTGGGCGTCACCGAATTCGAAGCCCGCTACGGAACGCTCCCCCCAGCCTCGCAGCGTTGGGACCGTATCCTCCAGGCATCAGGGATGAAAAGGGCCAAACCGTCCCCTACTTCAGCTCAAACACCGGATCAGGCGTCTTTGCATGCATTCGCCGATTCGCTGGAGCGTGACCTTGATGCGCCCAGCCCAATGCACGAGGGAGATCAGACGCGGGCAAGCAGCCGTAAACGGTCCCGATCGGATCGTGCTGTCACCGGCCCCTCCACACAGCAATCTTTCGAGGTGCGCGTTCCCGAACAGCAAGATGCGCTGCATTTGCCCCTCAGCTGGAGGGTAAAACGCCCGCGTACCAGGATCGGGGGCGGCCTCCCGGATCCTGGTACGCCCATCGCTGCCGACCTGGCAGCGTCCAGCACCGTGATGTGGGAACAAGATGCGGCCCCCTTCGCAGGGGCAGCGGATGATTTCCCGGCATTCAACGAAGAGGAGCTCGCATGGTTGATGGAGCTATTGCCTCAGTCAGGCTCAGTCGGAGGGACGATCTGA

**Gin-AvrΔ74**

MLIGYVRVSTNDQNTDLQRNALVCAGCEQIFEDKLSGTRTDRPGLKRALKRLQKGDTLVVWKLDRLGRSMKHLISLVGELRERGINFRSLTDSIDTSSPMGRFFFYVMGALAEMERELIIERTMAGLAAARNKGRIGGRPRKSGSGSPRQFDPSLLDTSLLDSMPAVGTPHTAAAPAECDEVQSGLRAADDPPPTVRVAVTAARPPRAKPAPRRRAAQPSDASPAAQVDLRTLGYSQQQQEKIKPKVRSTVAQHHEALVGHGFTHAHIVALSQHPAALGTVAVTYQDIIRALPEATHEDIVGVGKQWSGARALEALLTEAGELRGPPLQLDTGQLLKIAKRGGVTAVEAVHAWRNALTGAPLNLTPDQVVAIASNIGGKQALETVQRLLPVLCQDHGLTPDQVVAIASHGGGKQALETVQRLLPVLCQDHGLTPDQVVAIASNIGGKQALETVQRLLPVLCQAHGLTPDQVVAIASNIGGKQALETVQRLLPVLCQDHGLTPAQVVAIASNSGGKQALETVQRLLPVLCQDHGLTPDQVVAIASHDGGKQALETLQRLLPVLCQDHGLTPDQVVAIANNNGGKQALETLQRLLPVLCQDHGLTPDQVVAIASHDGGKQALETVQRLLPVLCQDHGLTPDQVVAIASHDGGKQALETVQRLLPVLCQDHGLTPAQVVAIASHDGGKQALETVQRLLPVLCQDHGLTPDQVVAIASNSGGKQALETVQRLLPVLCQDHGLTPDQVVAIASNGGKQALETVQRLLPVLCQDHGLTPDQVVAIASNGGKQALETVQRLLPVQRLLPVLCQDHGLTQDQVVAIASHDGGKQALETVQRLLPVLCQDHGLTPDQVVAIASHDGGKQALETVQRLLPVLCQDHGLTPDQVVAIASNSGGKQALETVQRLLPVLCQDHGLTPDQVVAIASNSGGKQALETVQRLLPVLCQDHGLTPDQVVAIASNNGGKQALETVQRLLPVLCQDHGLTPDQVVAIANNNGGKQALETVQRLLPVLCQDHGLTPAQVVAIASNIGGKQALETVQRLLPVLCQDHGLTLDQVVAIASNGGSKQALETVQRLLPVLCQDHGLTPDQVVAIANNNGGKQALETVQRLLPVLCQDHGLTPDQVVAIASNIGGKQALETVQRLLPVLCQDHGLTLDQVVAIASNGGKQALETVQRLLPVLCQDHGLTPNQVVAIASNSGGKQALETVQRLLPVLCQDHGLTPNQVVAIASNGGKQALESIVAQLSRPDPALAALTNDHLVALACLG

**Gin-AvrΔ87**

MLIGYVRVSTNDQNTDLQRNALVCAGCEQIFEDKLSGTRTDRPGLKRALKRLQKGDTLVVWKLDRLGRSMKHLISLVGELRERGINFRSLTDSIDTSSPMGRFFFYVMGALAEMERELIIERTMAGLAAARNKGRIGGRPRKSGSGSPDSMPAVGTPHTAAAPAECDEVQSGLRAADDPPPTVRVAVTAARPPRAKPAPRRRAAQPSDASPAAQVDLRTLGYSQQQQEKIKPKVRSTVAQHHEALVGHGFTHAHIVALSQHPAALGTVAVTYQDIIRALPEATHEDIVGVGKQWSGARALEALLTEAGELRGPPLQLDTGQLLKIAKRGGVTAVEAVHAWRNALTGAPLNLTPDQVVAIASNIGGKQALETVQRLLPVLCQDHGLTPDQVVAIASHGGGKQALETVQRLLPVLCQDHGLTPDQVVAIASNIGGKQALETVQRLLPVLCQAHGLTPDQVVAIASNIGGKQALETVQRLLPVLCQDHGLTPAQVVAIASNSGGKQALETVQRLLPVLCQDHGLTPDQVVAIASHDGGKQALETLQRLLPVLCQDHGLTPDQVVAIANNNGGKQALETLQRLLPVLCQDHGLTPDQVVAIASHDGGKQALETVQRLLPVLCQDHGLTPDQVVAIASHDGGKQALETVQRLLPVLCQDHGLTPAQVVAIASHDGGKQALETVQRLLPVLCQDHGLTPDQVVAIASNSGGKQALETVQRLLPVLCQDHGLTPDQVVAIASNGGKQALETVQRLLPVLCQDHGLTPDQVVAIASNGGKQALETVQRLLPVQRLLPVLCQDHGLTQDQVVAIASHDGGKQALETVQRLLPVLCQDHGLTPDQVVAIASHDGGKQALETVQRLLPVLCQDHGLTPDQVVAIASNSGGKQALETVQRLLPVLCQDHGLTPDQVVAIASNSGGKQALETVQRLLPVLCQDHGLTPDQVVAIASNNGGKQALETVQRLLPVLCQDHGLTPDQVVAIANNNGGKQALETVQRLLPVLCQDHGLTPAQVVAIASNIGGKQALETVQRLLPVLCQDHGLTLDQVVAIASNGGSKQALETVQRLLPVLCQDHGLTPDQVVAIANNNGGKQALETVQRLLPVLCQDHGLTPDQVVAIASNIGGKQALETVQRLLPVLCQDHGLTLDQVVAIASNGGKQALETVQRLLPVLCQDHGLTPNQVVAIASNSGGKQALETVQRLLPVLCQDHGLTPNQVVAIASNGGKQALESIVAQLSRPDPALAALTNDHLVALACLG

**Gin-AvrΔ120**

MLIGYVRVSTNDQNTDLQRNALVCAGCEQIFEDKLSGTRTDRPGLKRALKRLQKGDTLVVWKLDRLGRSMKHLISLVGELRERGINFRSLTDSIDTSSPMGRFFFYVMGALAEMERELIIERTMAGLAAARNKGRIGGRPRKSGSGSTVRVAVTAARPPRAKPAPRRRAAQPSDASPAAQVDLRTLGYSQQQQEKIKPKVRSTVAQHHEALVGHGFTHAHIVALSQHPAALGTVAVTYQDIIRALPEATHEDIVGVGKQWSGARALEALLTEAGELRGPPLQLDTGQLLKIAKRGGVTAVEAVHAWRNALTGAPLNLTPDQVVAIASNIGGKQALETVQRLLPVLCQDHGLTPDQVVAIASHGGGKQALETVQRLLPVLCQDHGLTPDQVVAIASNIGGKQALETVQRLLPVLCQAHGLTPDQVVAIASNIGGKQALETVQRLLPVLCQDHGLTPAQVVAIASNSGGKQALETVQRLLPVLCQDHGLTPDQVVAIASHDGGKQALETLQRLLPVLCQDHGLTPDQVVAIANNNGGKQALETLQRLLPVLCQDHGLTPDQVVAIASHDGGKQALETVQRLLPVLCQDHGLTPDQVVAIASHDGGKQALETVQRLLPVLCQDHGLTPAQVVAIASHDGGKQALETVQRLLPVLCQDHGLTPDQVVAIASNSGGKQALETVQRLLPVLCQDHGLTPDQVVAIASNGGKQALETVQRLLPVLCQDHGLTPDQVVAIASNGGKQALETVQRLLPVQRLLPVLCQDHGLTQDQVVAIASHDGGKQALETVQRLLPVLCQDHGLTPDQVVAIASHDGGKQALETVQRLLPVLCQDHGLTPDQVVAIASNSGGKQALETVQRLLPVLCQDHGLTPDQVVAIASNSGGKQALETVQRLLPVLCQDHGLTPDQVVAIASNNGGKQALETVQRLLPVLCQDHGLTPDQVVAIANNNGGKQALETVQRLLPVLCQDHGLTPAQVVAIASNIGGKQALETVQRLLPVLCQDHGLTLDQVVAIASNGGSKQALETVQRLLPVLCQDHGLTPDQVVAIANNNGGKQALETVQRLLPVLCQDHGLTPDQVVAIASNIGGKQALETVQRLLPVLCQDHGLTLDQVVAIASNGGKQALETVQRLLPVLCQDHGLTPNQVVAIASNSGGKQALETVQRLLPVLCQDHGLTPNQVVAIASNGGKQALESIVAQLSRPDPALAALTNDHLVALACLG

**Gin-AvrΔ120***

MLIGYVRVSTNDQNTDLQRNALVCAGCEQIFEDKLSGTRTDRPGLKRALKRLQKGDTLVVWKLDRLGRSMKHLISLVGELRERGINFRSLTDSIDTSSPMGRFFFYVMGALAEMERELIIERTMAGLAAARNKGRIGGRPRKSGSGSTVRVAVTAARPPHAVAGPAAQVDLRTLGYSQQQQEKIKPKVRSTVAQHHEALVGHGFTHAHIVALSQHPAALGTVAVTYQDIIRALPEATHEDIVGVGKQWSGARALEALLTEAGELRGPPLQLDTGQLLKIAKRGGVTAVEAVHAWRNALTGAPLNLTPDQVVAIASNIGGKQALETVQRLLPVLCQDHGLTPDQVVAIASHGGGKQALETVQRLLPVLCQDHGLTPDQVVAIASNIGGKQALETVQRLLPVLCQAHGLTPDQVVAIASNIGGKQALETVQRLLPVLCQDHGLTPAQVVAIASNSGGKQALETVQRLLPVLCQDHGLTPDQVVAIASHDGGKQALETLQRLLPVLCQDHGLTPDQVVAIANNNGGKQALETLQRLLPVLCQDHGLTPDQVVAIASHDGGKQALETVQRLLPVLCQDHGLTPDQVVAIASHDGGKQALETVQRLLPVLCQDHGLTPAQVVAIASHDGGKQALETVQRLLPVLCQDHGLTPDQVVAIASNSGGKQALETVQRLLPVLCQDHGLTPDQVVAIASNGGKQALETVQRLLPVLCQDHGLTPDQVVAIASNGGKQALETVQRLLPVQRLLPVLCQDHGLTQDQVVAIASHDGGKQALETVQRLLPVLCQDHGLTPDQVVAIASHDGGKQALETVQRLLPVLCQDHGLTPDQVVAIASNSGGKQALETVQRLLPVLCQDHGLTPDQVVAIASNSGGKQALETVQRLLPVLCQDHGLTPDQVVAIASNNGGKQALETVQRLLPVLCQDHGLTPDQVVAIANNNGGKQALETVQRLLPVLCQDHGLTPAQVVAIASNIGGKQALETVQRLLPVLCQDHGLTLDQVVAIASNGGSKQALETVQRLLPVLCQDHGLTPDQVVAIANNNGGKQALETVQRLLPVLCQDHGLTPDQVVAIASNIGGKQALETVQRLLPVLCQDHGLTLDQVVAIASNGGKQALETVQRLLPVLCQDHGLTPNQVVAIASNSGGKQALETVQRLLPVLCQDHGLTPNQVVAIASNGGKQALESIVAQLSRPDPALAALTNDHLVALACLG

**Gin-AvrΔ147**

MLIGYVRVSTNDQNTDLQRNALVCAGCEQIFEDKLSGTRTDRPGLKRALKRLQKGDTLVVWKLDRLGRSMKHLISLVGELRERGINFRSLTDSIDTSSPMGRFFFYVMGALAEMERELIIERTMAGLAAARNKGRIGGRPRKSGSGSPASPAAQVDLRTLGYSQQQQEKIKPKVRSTVAQHHEALVGHGFTHAHIVALSQHPAALGTVAVTYQDIIRALPEATHEDIVGVGKQWSGARALEALLTEAGELRGPPLQLDTGQLLKIAKRGGVTAVEAVHAWRNALTGAPLNLTPDQVVAIASNIGGKQALETVQRLLPVLCQDHGLTPDQVVAIASHGGGKQALETVQRLLPVLCQDHGLTPDQVVAIASNIGGKQALETVQRLLPVLCQAHGLTPDQVVAIASNIGGKQALETVQRLLPVLCQDHGLTPAQVVAIASNSGGKQALETVQRLLPVLCQDHGLTPDQVVAIASHDGGKQALETLQRLLPVLCQDHGLTPDQVVAIANNNGGKQALETLQRLLPVLCQDHGLTPDQVVAIASHDGGKQALETVQRLLPVLCQDHGLTPDQVVAIASHDGGKQALETVQRLLPVLCQDHGLTPAQVVAIASHDGGKQALETVQRLLPVLCQDHGLTPDQVVAIASNSGGKQALETVQRLLPVLCQDHGLTPDQVVAIASNGGKQALETVQRLLPVLCQDHGLTPDQVVAIASNGGKQALETVQRLLPVQRLLPVLCQDHGLTQDQVVAIASHDGGKQALETVQRLLPVLCQDHGLTPDQVVAIASHDGGKQALETVQRLLPVLCQDHGLTPDQVVAIASNSGGKQALETVQRLLPVLCQDHGLTPDQVVAIASNSGGKQALETVQRLLPVLCQDHGLTPDQVVAIASNNGGKQALETVQRLLPVLCQDHGLTPDQVVAIANNNGGKQALETVQRLLPVLCQDHGLTPAQVVAIASNIGGKQALETVQRLLPVLCQDHGLTLDQVVAIASNGGSKQALETVQRLLPVLCQDHGLTPDQVVAIANNNGGKQALETVQRLLPVLCQDHGLTPDQVVAIASNIGGKQALETVQRLLPVLCQDHGLTLDQVVAIASNGGKQALETVQRLLPVLCQDHGLTPNQVVAIASNSGGKQALETVQRLLPVLCQDHGLTPNQVVAIASNGGKQALESIVAQLSRPDPALAALTNDHLVALACLG

**GinAvr15Δ128-synthetic protein**:

MLIGYVRVSTNDQNTDLQRNALVCAGCEQIFEDKLSGTRTDRPGLKRALKRLQKGDTLVVWKLDRLGRSMKHLISLVGELRERGINFRSLTDSIDTSSPMGRFFFYVMGALAEMERELIIERTMAGLAAARNKGRIGGRPRKSGSGSPALRPPRAKPAPRRRAAQPSDASPAAQVDLRTLGYSQQQQEKIKPKVRSTVAQHHEALVGHGFTHAHIVALSQHPAALGTVAVTYQHIITALPEATHEDIVGVGKQWSGARALEALLTDAGELRGPPLQLDTGQLVKIAKRGGVTAMEAVHASRNALTGAPLNLTPDQVVAIASNIGGKQALETVQRLLPVLCQDHGLTPDQVVAIASNGGGKQALETVQRLLPVLCQDHGLTPDQVVAIASNIGGKQALETVQRLLPVLCQDHGLTPDQVVAIASNIGGKQALETVQRLLPVLCQDHGLTPDQVVAIASNIGGKQALETVQRLLPVLCQDHGLTPDQVVAIASHDGGKQALETVQRLLPVLCQDHGLTPDQVVAIASHDGGKQALETVQRLLPVLCQDHGLTPDQVVAIASHDGGKQALETVQRLLPVLCQDHGLTPDQVVAIASHDGGKQALETVQRLLPVLCQDHGLTPDQVVAIASHDGGKQALETVQRLLPVLCQDHGLTPDQVVAIASNGGGKQALETVQRLLPVLCQDHGLTPDQVVAIASHDGGKQALETVQRLLPVLCQDHGLTPDQVVAIASHDGGKQALETVQRLLPVLCQDHGLTPDQVVAIASNIGGKQALETVQRLLPVLCQDHGLTPDQVVAIASNIGGKQALESIVAQLSRPDPALAALTNDHLVALACLGPKKKRKV

**Gin-Avr15Δ128-synthetic DNA**:

ATGCTGATTGGCTATGTAAGGGTATCAACAAATGACCAGAATACAGACCTGCAACGAAACGCTCTTGTTTGTGCAGGATGTGAACAAATATTTGAAGATAAATTAAGCGGAACAAGGACAGACCGACCGGGATTAAAACGCGCTTTAAAGCGCCTTCAAAAAGGTGACACACTGGTTGTCTGGAAACTGGATCGCCTCGGGCGAAGCATGAAACATTTGATTTCTCTCGTAGGGGAATTACGAGAGCGAGGGATTAATTTTCGCAGTCTTACTGACAGTATTGATACGTCATCTCCAATGGGGCGTTTTTTCTTCTACGTTATGGGTGCCCTGGCTGAAATGGAACGAGAACTAATTATCGAGCGAACGATGGCTGGACTTGCTGCCGCCAGAAATAAAGGCCGTATTGGAGGTCGCCCGCGTAAATCGGGGTCTGGATCCCCCGCGCGGCCGCCGCGCGCCAAGCCGGCCCCGCGACGGCGTGCTGCGCAACCCTCCGACGCTTCGCCGGCCGCGCAGGTGGATCTACGCACGCTCGGCTACAGTCAGCAGCAGCAAGAGAAGATCAAACCGAAGGTGCGTTCGACAGTGGCGCAGCACCACGAGGCACTGGTGGGCCATGGGTTTACACACGCGCACATCGTTGCGCTCAGCCAACACCCGGCAGCGTTAGGGACCGTCGCTGTCACGTATCAGCACATAATCACGGCGTTGCCAGAGGCGACACACGAAGACATCGTTGGCGTCGGCAAACAGTGGTCCGGCGCACGCGCCCTGGAGGCCTTGCTCACGGATGCGGGGGAGTTGAGAGGTCCGCCGTTACAGTTGGACACAGGCCAACTTGTGAAGATTGCAAAACGTGGCGGCGTGACCGCAATGGAGGCAGTGCATGCATCGCGCAATGCACTGACGGGTGCCCCCCTGGAGCTGACTCCGGACCAAGTGGTGGCTATCGCCAGCAACATTGGCGGCAAGCAAGCGCTCGAAACGGTGCAGCGGCTGTTGCCGGTGCTGTGCCAGGACCATGGCCTGACTCCGGACCAAGTGGTGGCTATCGCCAGCAACGGTGGCGGCAAGCAAGCGCTCGAAACGGTGCAGCGGCTGTTGCCGGTGCTGTGCCAGGACCATGGCCTGACTCCGGACCAAGTGGTGGCTATCGCCAGCAACATTGGCGGCAAGCAAGCGCTCGAAACGGTGCAGCGGCTGTTGCCGGTGCTGTGCCAGGACCATGGCCTGACTCCGGACCAAGTGGTGGCTATCGCCAGCAACATTGGCGGCAAGCAAGCGCTCGAAACGGTGCAGCGGCTGTTGCCGGTGCTGTGCCAGGACCATGGCCTGACTCCGGACCAAGTGGTGGCTATCGCCAGCAACATTGGCGGCAAGCAAGCGCTCGAAACGGTGCAGCGGCTGTTGCCGGTGCTGTGCCAGGACCATGGCCTGACTCCGGACCAAGTGGTGGCTATCGCCAGCCACGATGGCGGCAAGCAAGCGCTCGAAACGGTGCAGCGGCTGTTGCCGGTGCTGTGCCAGGACCATGGCCTGACTCCGGACCAAGTGGTGGCTATCGCCAGCCACGATGGCGGCAAGCAAGCGCTCGAAACGGTGCAGCGGCTGTTGCCGGTGCTGTGCCAGGACCATGGCCTGACTCCGGACCAAGTGGTGGCTATCGCCAGCCACGATGGCGGCAAGCAAGCGCTCGAAACGGTGCAGCGGCTGTTGCCGGTGCTGTGCCAGGACCATGGCCTGACTCCGGACCAAGTGGTGGCTATCGCCAGCCACGATGGCGGCAAGCAAGCGCTCGAAACGGTGCAGCGGCTGTTGCCGGTGCTGTGCCAGGACCATGGCCTGACTCCGGACCAAGTGGTGGCTATCGCCAGCCACGATGGCGGCAAGCAAGCGCTCGAAACGGTGCAGCGGCTGTTGCCGGTGCTGTGCCAGGACCATGGCCTGACCCCGGACCAAGTGGTGGCTATCGCCAGCAACGGTGGCGGCAAGCAAGCGCTCGAAACGGTGCAGCGGCTGTTGCCGGTGCTGTGCCAGGACCATGGCCTGACTCCGGACCAAGTGGTGGCTATCGCCAGCCACGATGGCGGCAAGCAAGCGCTCGAAACGGTGCAGCGGCTGTTGCCGGTGCTGTGCCAGGACCATGGCCTGACTCCGGACCAAGTGGTGGCTATCGCCAGCCACGATGGCGGCAAGCAAGCGCTCGAAACGGTGCAGCGGCTGTTGCCGGTGCTGTGCCAGGACCATGGCCTGACCCCGGACCAAGTGGTGGCTATCGCCAGCAACATTGGCGGCAAGCAAGCGCTCGAAACGGTGCAGCGGCTGTTGCCGGTGCTGTGCCAGGACCATGGCCTGACCCCGGACCAAGTGGTGGCTATCGCCAGCAACATTGGCGGCAAGCAAGCGCTCGAAAGCATTGTGGCCCAGCTGAGCCGGCCTGATCCGGCGTTGGCCGCGTTGACCAACGACCACCTCGTCGCCTTGGCCTGCCTCGGCCCCAAGAAGAAGCGCAAGGTGTAG
